# ABCG5 gene responses to treadmill running with or without administration of Pistachio atlantica in female rats

**Published:** 2014-03

**Authors:** Abbass Ghanbari-Niaki, Navabeh Zare-Kookandeh, Asghar Zare-Kookandeh

**Affiliations:** 1 Exercise Biochemistry Division, Faculty of Physical Education and Sport Science, University of Mazandaran, Baboulsar, Mazandaran, Iran; 2 Tehran Heart Center, Tehran University of Medical Sciences, Tehran, Iran

**Keywords:** ABCG5, ABC transporters, Female rats, *Pistachia atlantica*, Treadmill exercise

## Abstract

***Objective(s): ***ABC transporters comprise a large family of transmembrane proteins that use the energy provided by ATP hydrolysis to translocate a variety of substrates across biological membranes. All members of the human ABCG subfamily, except for ABCG2, are cholesterol-transporter. The aim of this study was to determine the liver, the small intestine and kidney ABCG5 relative gene expression in response to treadmill-running training in female rats.

***Materials and Methods: ***Twenty Wistar rats (6-8 weeks old and 125-135 g weight) were used. Animals were randomly assigned to saline-control (SC), saline-training (ST), and Baneh-control (BC), and Baneh-training (BT) groups. Training groups did the exercise on a motor-driven treadmill at 25 m/min (0% grade) for 60 min/day for eight weeks (5 days/week). Rats were fed orally, with Baneh extraction and saline for six weeks. The two-way ANOVA was employed for statistical analysis. ABCG5 relative gene expression was detected by Real-time PCR method.

***Results:*** The current findings indicate that the Baneh-treated tissues had significantly lower levels of ABCG5 gene expression in the liver, small intestine, and kidneys (*P*< 0.001, *P*< 0.003, *P*< 0.001, respectively), when compared with saline-treated tissues. However, a higher level of gene expression was observed in exercise groups. A lower level of HDL-c but not triglyceride (TG) and total cholesterol (TC) levels were found in Baneh-treated animals at rest.

***Conclusion: ***Exercise training increases ABCG5 relative gene expression in the liver, small intestine and kidney tissues; therefore exercise training may adjust the reduction of ABCG5 relative gene expression in Baneh-training group.

## Introduction

ABC transporters comprise a large family of transmembrane proteins that use the energy provided by ATP hydrolysis to translocate a variety of substrates across biological membranes (1). The members of this family have been categorized into seven subfamilies (ABC-A-G). ABCG has several members such as ABCG 1, ABCG 2, ABCG 3, ABCG 4, ABCG 5, ABCG 8, ABCG 11, ABCG 12 and ABCG 26 (2-5). All members of the human ABCG subfamily, except for ABCG2/BCRP, function as cholesterol transporters (6). ABC transporters are involved in the biliary secretion of bile acids (ABCB11), phospholipids (ABCB4) and cholesterol (ABCG5/G8) (7). ABCG5 and ABCG8 are half-transporters that could make a heterodimer to become a functional transporter (8-10). Both proteins are expressed at a high level in the liver and intestine and at lower levels in the colon (11-15). The 2 adenosine triphosphate (ATP)-binding cassette (ABC) half-transporters ABCG5 and ABCG8 are expressed in the mucosa cells and the canalicular membrane, and they resecrete sterols, especially absorbed plant sterols, back into the intestinal lumen and from the liver into bile (16, 17).

Respective mutations have been identified as the disease substrate for sitosterolemia (7). Sitosterolemic patients are highly sensitive to dietary cholesterol and become markedly hypercholesterolemic when fed with a high-cholesterol diet (18-21). Along with other dietary sterols, sitosterolemia patients absorb a greater fraction of dietary cholesterol and excrete less cholesterol into the bile as compared with normal subjects, resulting in a hypercholesterolemia with elevated apolipoprotein B levels but with reduced hepatic cholesterol biosynthesis (22-24). The high plasma cholesterol levels are associated with severe premature atherosclerosis (19, 25, 26). The elevated level of plant sterols may represent an independent risk factor for the development of atherosclerosis (27, 28).

Over the last few decades, in most countries the rate of people being treated with alternative therapy, particularly dietary supplements and herbal, has increased (29-38). Herbal medicine has fewer side effects and now is also recommended by the World Health Organization (WHO). The effects of dietary supplement on healthy men have shown that walnuts oil could decrease total blood cholesterol (39). The effect of silymarin on animals that were fed with high-fat diets showed that this plant has a positive effect on plasma lipoproteins profile (40). It has been reported that several factors (nutrients, fasting, and physical stress) could affect gene expression. Sobolova *et al *reported that silymarin has a positive feedback on ABC Transporters (41). In recent years, many researches have been done on the ABC family. One of these researches was carried out by Ghanbari-Niaki *et al*, in which the effect of 6 week endurance exercise on ABCA1 gene expression was investigated in rats. They reported that ABCA1 gene expression increases in rat liver tissue and the plasma levels of high density lipoprotein- cholesterol (HDL-C), Pre-β HDL and lecithin cholesterol acyltransferase (LCAT) significantly increased (42). Khabazian *et al* showed that 12 weeks of aerobic exercise, increased mRNA expressions of ABCA1 gene expression in rats small intestine (43). However, no study has examined the effect of exercise on ABCG5 gene expression. Also, there is lack of information on the effect of *Pistachio atlantica* on tissue ABCG5 expression. The total amount of essential oil obtained from *P. atlantica* is higher than any other species of the genus *Pistachio* (44). Also, *P. atlantica* has anti-inflammatory effect. In this study, we investigated the liver, small intestine and kidney ABCG5 gene expression, as well as plasma HDL-C, TC and TG concentrations after 8 weeks of treadmill running program and Baneh extraction treatment in wild type female rats.

## Materials and Methods

***Plant material***

The matured fruit samples of *P. atlantica* (Baneh) were collected from the agriculture fields on Maibod city in Yazd, Iran, and stored at –18°C until used. Plant material was identified by experts in the Herbarium of Biology Department of Faculty of Basic Sciences, University of Mazandaran, and Baboulsar, Iran.

***Preparation of the extracts***

The extracts were prepared by maceration (72 hr) of the coarsely powdered of whole parts of *P. atlantica*, with 150 ml tap water for 45 min at room temperature and were filtered twice through filter paper. The volume of the filtered solution was increased to 100 ml with tap water so that one ml was equivalent to 100 mg of the preparatory material (45). The freshly prepared extracts were cooled and immediately used in the experiments. To the herbalists’ recommendation, distilled water was not used for the extraction. After training, 100 mg/kg liquid extraction of Baneh was orally administered to the Baneh groups and the same amount of saline was fed to saline groups.

***Preparation of the GC/MS analyses***

The whole ripped and dried fruits of *P. atlantica* were grounded by an electronic grinder (Moulinex, France) to a fine powder. A part of the powdered plant was macerated with n-hexane (Merck, USA) for 72 hr at room temperature, extracted by soxhlet extractor (Schott Duran, Germany), with collaboration of the Faculty of Chemistry, University of Mazandaran, and evaporated by a rotary evaporator (D-91126 Schwabach, Type Heizbad WB eco, Ser. No: 060819780, Germany). Chromatographic analysis was carried out on *Hewlett Packard (HP) *devices (6890 series GC-MS apparatus ) combined only with a front detector FID and with two capillary Columns (Capillary Column 1: Model Number: HP 19091S-633- HP-1MS, and Capillary Column 2: Model Number: Agilent 19091J-133-HP-5) (Agilent Technologies, Canada). The fatty acid components of the *P. atlantica* extracts were determined by library search software from The Wiley/NBS Registry Mass Spectral Data and in-house “BASER Library of Fatty Acid Constituents”.

***Animals***

All experiments that involved the animals were conducted according to the policy of the Iranian convention for the protection of vertebrate animals used for experimental and other scientific purposes; and the protocol was approved by the Ethics Committee of the Sciences, University of Mazandaran (UMZ) and Babol University of Medical Sciences, Mazandaran, Iran. Twenty Wistar female rats (6-8 weeks old, 125-135 g weight) were acquired from Pasteur Institute (Amol, Mazandaran, Iran) and maintained in the Central Animal House of Faculty of Physical Education and Sports Science of UMZ. Five rats were housed in each cage (46-L volume) with a 12 hr: 12 hr light-dark cycle. Temperature was maintained at 22°C ± 1.4°C. Diets (a pellet form) and water were provided *ad libitum*. Animals were randomly assigned to control (n= 10) and training (n= 10) groups. Rats were divided further into saline-control (SC), saline-training (ST), and Baneh-control (BC), and Baneh-training (BT). The control group remained sedentary, whereas the training group underwent a moderate running exercise program.

***Exercise training protocol***

At first, the animals were familiarized with the rat treadmill apparatus, every day for 4 days. The exercise group was trained for 8 weeks using the same training methods as previously described (42, 43).

**Table 1:** Oligonucleotide primer sequences and real-time PCR amplification parameters

Gene	Forward and reverse primer sequences	Annealing temperature (°C)	Amplicon size (bp)	Gene accession no.
ABCG5	F:5׳-AGGCTCAGTTACAGGCTCAGAG-3׳ R:5׳-GTCCCACTTCTGCTGGCATGAT-3׳	60°C	118	AF312714
β-actin	F:5׳-TATCGGCAATGAGCGGTTCC-3׳ R:5׳-AGCACTGTGTTGGCATAGAGG-3׳	60°C	145	NM_031144

The rats run at 25 m/min for 60 min, 5 day/week. The animals were sacrificed 72 hr after the last exercise session. Food but not water was removed from the rat cages 4 hr before the sacrifices. The estrous cycle was determined in intact female rats by taking vaginal smears each morning by vaginal lavage. Smears were analyzed under a microscope to determine the type of cells present and the stage of the estrous cycle (46). Only female rats showing at least two consecutive 4- or 5-day estrous cycles were used. The establishment of the estrous cycle in each female rat was used to select the day of the experiment, and the estrous cycle stage was confirmed by vaginal smear (47).

***Tissue biopsies***

Seventy-two hr after the last training session, rats were anesthetized with intra peritoneal administration of a mixture of ketamine (30–50 mg/ kg body weight) and xylazine (3–5 mg/kg body weight). The Liver, the small intestine and the kidney were excised, cleaned, divided into two pieces, washed in ice-cold saline, and immediately frozen in liquid nitrogen and stored at −80°C until RNA extraction.

***RNA,*** ***cDNA synthesis and real-time PCR***

Total RNA was extracted from 80 to 100 mg of tissue, using RNA purification kits (AccuZol, Bioneer, Republic of Korea). Complementary DNA (cDNA) was extended from 1 µl oligo-(dt)_18_ primers (0.25 μg per reaction)using cDNA synthesis kit (AccuPower RT PreMix, Bioneer, Republic of Korea) according to the manufacturer instructions. Real-time quantitative PCR was performed by using QuantiFast SYBR Green PCR Kit (Catalogue Number 204052, Qiagen, GmbH, Germany). Fifteen microliter (15 μl) reaction containing 0.5 μl single-strand cDNA, 7.5 μl Master Mix, 1 μl of the each forward and reverse primers (5 pmol/μl) and 5 μl dH_2_O. The primers forABCG5 and β-actin (as normalizer) were taken from Hongwei et al, 2003 and Gao &Yuan, 2010, respectively) (Table 1) (34, 35). Real-time PCR reactions were performed using the Rotor Gene 3000 real time PCR system from Corbett using following program: step1: 95°C for 5 min and step 2: 40 cycle of 95°C for 10 sec and 60°C for 30 sec. The last heating step in phase 2 was carried out for generation of a melting curve of the product. The amplicons were melted at the rate of 0.1°C/sec to generate the high resolution melting profile. Product specificity was confirmed in the initial experiments by 3% agarose gel electrophoresis and melting curve analysis.

***Lipids and lipoproteins measurements***

Plasma concentration of high density lipoprotein cholesterol (HDL) was determined by direct Immuno method (HDL-C Immuno FS, Pars Azmoun, Tehran, Iran), the Intra-assay coefficient of variation and sensitivity of the method were 1.2% and 0.03 mmol/l. Plasma concentration of total triglyceride (TG) was determined by enzymatic (GPO, glycerol-3-phosphate oxidase) colorimetric method (Pars Azmoun, Tehran, Iran), the Intra-assay coefficient of variation and sensitivity of the method were 2.2% and 1 mg/dl, respectively. Plasma concentration of total cholesterol (TC) was determined by enzymatic (CHOD-PAP, cholesterol oxidase-amino antipyrine) colorimetric method (Pars Azmoun, Tehran, Iran), the Intra-assay coefficient of variation and sensitivity of the method were 1.9% and 0.08 mmol/l, respectively.

***Statistical analysis***

The relative levels of mRNA were analyzed by the^ 2 –ΔΔCt^ method (48). The Kolmogorov-Smirnov test was used to determine the normality of distribution, and variables were found to be normally distributed. All results are expressed as means ± SEM. Statistical analysis were performed using ANOVA. Least significant difference *post hoc* test was used in the event of a significant difference at *P*= 0.05. All statistical analysis was performed with SPSS (Version 13; Chicago, IL).

## Results

GC-MS analysis shows that *P. atlantica* is composed of α-pinene (0.71%), limonen (0.54%), hexadecenoic acid (7.52%), palmitinic acid (28.86%), trans-oleic acid (49.28%), n-octadecanoic acid (3.87%), oleic acid (0.2%), 9-octadecenoic acid (Z) (0.18%), 3-pentadecyl-phenol (2.69%), phenol, 3-pentadecyl (0.84%), 3-pentadecyl- phenol (1.58%), 4, 5:9, 10-dibenzo-1, 3, 6, 8-tetraazat ricyclo [4. 4. 1. 1 (3, 8)] dodecane 3-butyl-thiophene-1, 1-dioxide 3-(2, 2-dideuterobutyl)-thiophene-1, 1- dioxide (0.86%), 4, 5: 9, 10-dibenzo-1, 3, 6, 8-tetraazat ricyclo [4. 4. 1. 1 (3, 8)] dodecane cinnamyl cinnamate (0.28%), (acetoxymethyl) methyl [(trimethylsilyl) methyl] silane cinnamyl cinnamate (0.34%), 2, 4-diphenyl glutaronitrile 2-methyl-4, 5-diphenyl-4, 5-dihydroo xazole cinnamyl cinnamate (0.36%), phenol, 3-pentadecyl- acetic acid, 4-methylphenyl ester acetic acid, 4-methylphenyl ester (0.62%), phenol, 3-pentyl (0.36%), ribitol, and pentaacetate N-propenyl-2-methoxy-6-methylbenzamide 6, 7-dimethoxyisatin (0.15%) (Table 2).

**Table 2:** The main components of the whole fruits of *Pistachio atlantica *(Baneh) evaluated by GC-MS analysis

RT [min]	Library/ ID	Area %	Ref	Qual
39.200 36.056	Elaidic acid (trans-9-Octadecenoic acid) (E-Oleic acid) Palmitinic acid	49.28 28.86	228773 195439	99 99
35.196	Hexadecenoic acid (Oleic acid ester)	7.52	192904 (245468)	97 87
39.452	Stearic acid	3.87	231330	99
43.967	Tyramine	2.69	32747	38
44.258	3-Pentadecyl-Phenol (Anacardol)	2.42	254859	83
8.079	Alpha-pinene	0.71	32180	96

**Figure 1:**
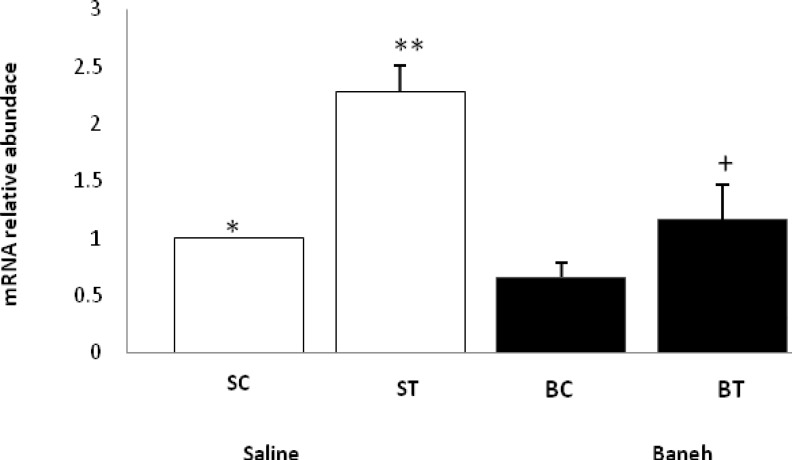
Real-time PCR of liver ABCG5 mRNA relative expression in saline- control (SC), saline-training (ST), Baneh-control (BC), and Baneh-training (BT) wild-type female rats. Wild-type female rats Data expressed as mean ± SEM. Each column is assigned to one group and 5 rats per group.*, SC vs ST, (*P*< 0.001) **, ST vs BT, (*P* < 0.001) +, BT vs BC, (*P*< 0.069)

**Figure 2:**
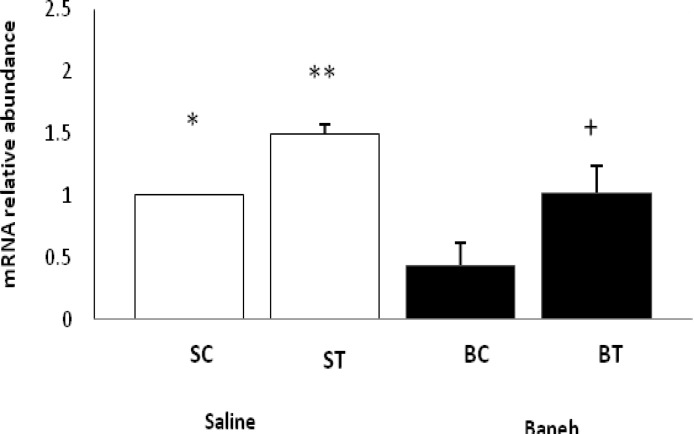
Real-time PCR of small intestine ABCG5 mRNA relative expression in saline- control (SC), saline-training (ST), Baneh-control (BC), and Baneh-training (BT) wild-type female rats. Wild-type female rats. Data expressed as mean ± SEM. Each column is assigned to one h group and 5 rat per group.*, SC vs ST, (*P* < 0.031) and SC vs BC, (*P* < 0.016) **, ST vs BT, (*P* < 0.047) +, BT vs BC , (*P* < 0.017)

**Figure 3:**
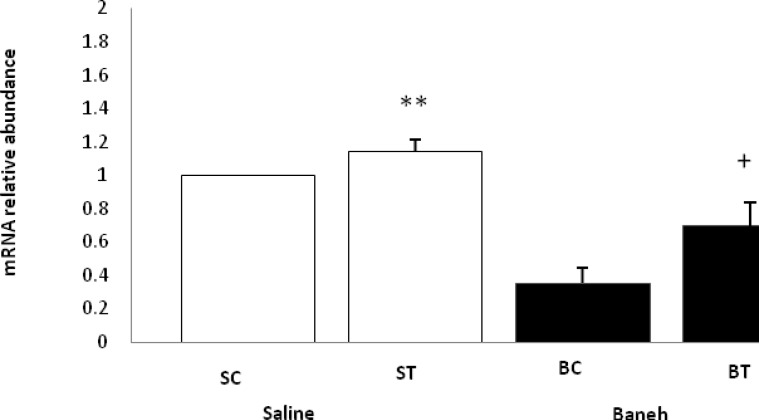
Real-time PCR kidney ABCG5 mRNA relative expression in saline- control (SC), saline-training (ST), Baneh-control (BC), and Baneh-training (BT) wild-type female rats. Wild-type female rats data expressed as mean ± SEM. Each column is assigned to one group and 5 rats per each group) **,ST vs BT, (*P *< 0.003) +, BT vs BC , (*P* < 0.015)

**Figure 4:**
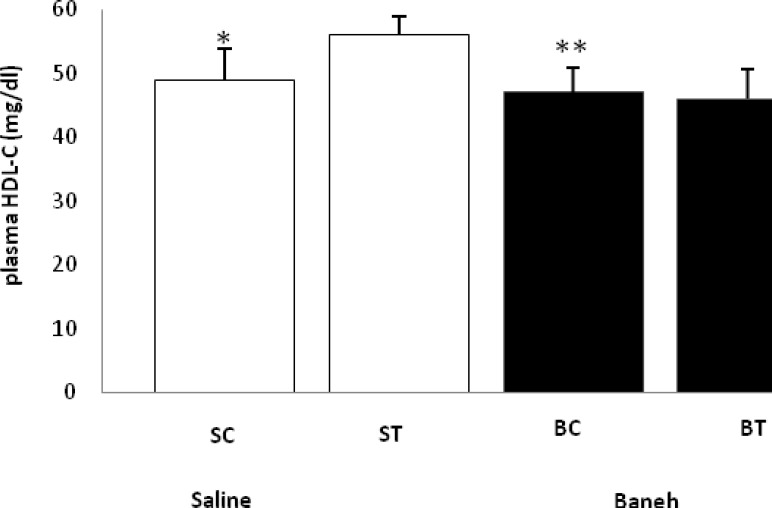
Plasma HDL-C concentration (mg/dl) in saline- control (SC), saline-training (ST), Baneh-control (BC), and Baneh-training (BT) wild-type female rats. Wild-type female rats Data expressed as mean Â± SEM. Each column is for each group and 5 rat per group.*, SC vs ST, (*P* < 0.04)

**Figure 5:**
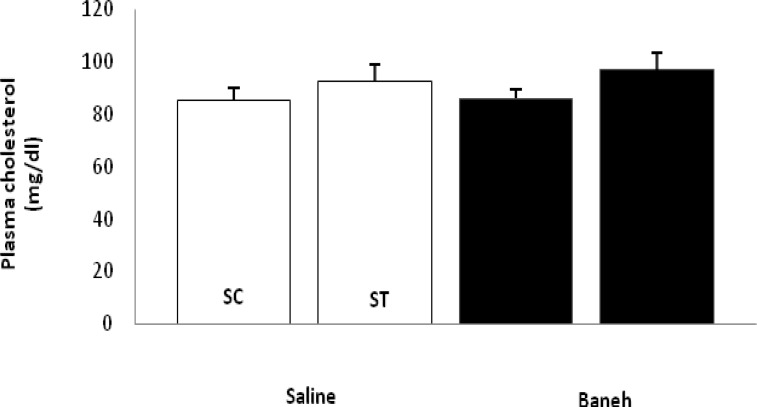
Plasma cholesterol concentration (mg/dl) in saline- control (SC), saline-training (ST), Baneh-control (BC), and Baneh-training (BT) wild-type female rats. Wild-type female rats data expressed as mean Â±SEM. Each column is for each group and 5 rat per group

**Figure 6:**
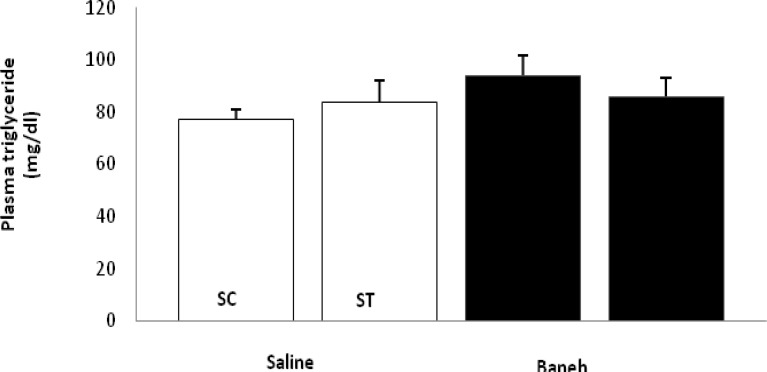
Plasma triglyceride concentration (mg/dl) in saline- control (SC), saline-training (ST), Baneh-control (BC), and Baneh-training (BT) wild-type female rats. Wild-type female rats data expressed as mean Â±SEM. Each column is for each group and 5 rat per group

ABCG5 relative gene expression in the liver, small intestine and kidney were determined in female rats. Data analysis revealed a significant difference in liver ABCG5 mRNA relative abundance between groups (F=14.50, *P*<0.001) (Figure 1). Using a suitable *post hoc* test, it was shown that the liver relative expression of ABCG5 was higher in ST group (2.2813±0.2285) when compared with other groups at the end of the experiment (Figure 1). Also, a significant difference was found in the small intestine relative mRNA expression of ABCG5 at the end of treadmill running program (F=8.158, *P*<0.003). In this regard, the ABCG5 mRNA relative abundance was lower in Baneh treated animals (1.0219±0.21305) when compared with saline-treated groups (1.4928±0.14083) (Figure 2). Considering the kidneys, the same results as the liver and intestine have been observed (F=17.61, *P*<0.001) (Figure 3). The levels of plasma HDL-c were significantly (*P*<0.05) lower in Baneh-treated animals when compare with their counterparts (Figure 4). There were no significant changes in plasma TG and TC concentrations, (Figure 5 and 6). Collectively, it should be noted that training groups, particularly saline-trained tissues had higher levels of ABCG5 gene expression than other groups.

## Discussion

The purpose of the study was to examine the effect of treadmill running program with and without administration of a liquid *P. atlantica* (Baneh) extraction on plasma lipid and tissues ABCG5 gene expression in female rats. The main finding of the current research was a lower tissues ABCG5 gene expression and plasma HDL-c concentration in Baneh-treated animals. The other important finding of this study was a higher ABCG5 gene expression in saline-trained tissues when compared to other treated groups. To our knowledge, this is the first report to demonstrate that Baneh extraction under our research condition reduced ABCG5 gene expression in different tissue. Also, here it was revealed that exercise enhanced ABCG gene expression in saline and Baneh -trained groups. 

ABCG5 as a member of ABC subfamily has been shown to promote biliary cholesterol secretion and reduce fractional absorption of dietary cholesterol. The expression of ABCG5 gene has been reported in the liver and small intestine (15), colon and choroid plexus (11-15). Our results are in agreement with previously reported data concerning ABCG5 detection and expression in the liver and small intestine. The effect of diet intervention on the expression of ABC family, particularly ABCG family members has been also studied by several investigators (49-53). Ghanbari-Niaki *et al *found that administration of a high dose of aqueous extraction of *P. atlantica* extraction reduced and increased the small intestine and the kidney ABCG8 expression, respectively (54). Considering the effect of physical exercise on ABC family gene expression, there are few studies in relation to the impact of different types of physical exercise on ABC family gene expression. In this regard, studies have been more focused on ABCA1 rather than ABCG family members (42, 43, 52, 54-57). In human subjects, a low intensity exercise training (walking) for 8 weeks has been shown to increase the levels of ABCA1 and ABCG1 expression in peripheral blood lymphocytes (56). A reduction in ABCG8 expression has also been found in trained kidney with Baneh treatment. Recently, Côté *et al *studied the effect of atherogenic diet (high fat/high cholesterol) and a progressive exercise training (15-26 m/min on 0%-10% slope, 15-60min/day, 5times/week for 6 weeks) on the liver and small intestine ABCG5 and ABCG8 gene expression. In this study, training increased ABCG5 and ABCG8 gene expression in trained liver and small intestine treated with standard not atherogenic diet (52). 

Thus, the present results are in agreement with above mentioned studies. The mechanism(s) via which Baneh supplementation and endurance training can influence the ABCG5 mRNA expression in female rat tissues are poorly understood. However, several possible mechanisms could be considered in relation to the endurance training and Baneh supplementation. A lower ABCG5 gene expression in Baneh-treated rats could be attributed to the ratio of cis-oleic/trans-oleic contents. Our GC-MS data analysis of the *P. atlantica* (Baneh) showed a lower Cis-form (18:1, cis-9) (7.52%) and higher trans form (eladic acid) (49.28%). We also found a considerable amount of palmitic acid (28.86%) and stearic acid (3.87%). Eladic acid, a trans isomer of oleic acid has been reported to increase CTEP activity, increase low-density lipoprotein (LDL) and decrease HDL concentration in man (58). Although we did not study some of nuclear receptors which are involved in cholesterol efflux such as peroxisome proliferator-activated receptor (PPAR), liver X receptor (LXR), and farnesoid X receptor (FXR), but it might be also possible that Baneh administration reduced ABCG5 expression via these nuclear receptors (52, 59, 60). Physical exercise has been shown to have impact on nuclear receptors. Butcher *et al *reported that LXRα, PPAR α and PPARγ were significantly increased following an 8 weeks of low intensity exercise program (56). Côté *et al *reported that feeding atherogenic diet reduced liver FXR (by about 70%) but did not change liver LXR gene expression and FXR expression in the small intestine (52). Zhang *et al *demonstrated that swimming training (5 day/week for 3 months) significantly increased PARα no PPARγ gene expression in OLETF rat liver (61). As demonstrated by Horowitz *et al* endurance training (70-85% HRmax, 35-45min/session, 4 days/week for 12-14 weeks) resulted in twofold increase in mean skeletal muscle PPARα protein compared with untrained skeletal muscle (62). It has been reported that adiponectin can accelerate RCT and its deficiency suppresses ABCA1 expression and Apo A-1 synthesis in the liver. Higher adiponectin concentrations were found in rat plasma and adipose tissue following the long-term treadmill running at different intensities (10 weeks, 20, 26 and 34 m/min) (63-66). Thus, it might be possible that endurance training and Baneh supplementation under our experimental condition were able to alter tissues ABCG5 expression via above mentioned mechanisms.

## Conclusion

In summary, this is the first study that demonstrates the effects of treadmill running program with or without administration of liquid *P. atlantica* (Baneh) extraction on ABCG5 gene expression in female rat tissues. The present data indicate that administration of Baneh extraction under our experimental conditions decreases ABCG5 gene expression in female rat tissues. Data also showed that exercise training program was able to enhance ABCG5 gene expression and relief a Baneh extraction-induced a reduction in ABCG5 gene expression to somewhat. This was accompanied with lower plasma HDL-c concentration. It seems that under our experimental conditions the suppression of ABCG5 might be attributed to the given dose (act as a high dose) and the amount of essential oil composition (such as a high trans-oleic acid compare to lower cis-oleic acid). The relive of Baneh-induced suppression of ABCG5 expression by endurance training might be also due to an enhancement of the fatty acids oxidation (as fuel for energy provision) and cholesterol metabolism during eight weeks of exercise program.
